# Hydroethanolic Extracts of *Haplopappus baylahuen* Remy and *Aloysia citriodora* Palau Have Bactericide Activity and Inhibit the Ability of *Salmonella* Enteritidis to Form Biofilm and Adhere to Human Intestinal Cells

**DOI:** 10.1155/2021/3491831

**Published:** 2021-01-27

**Authors:** Estefanía Elgueta, Javier Mena, Pedro A. Orihuela

**Affiliations:** ^1^Laboratorio de Inmunología de la Reproducción, Facultad de Química y Biología, Universidad de Santiago de Chile, Santiago, Chile; ^2^Centro de Nanociencia y Nanotecnología, Universidad de Santiago de Chile, Santiago, Chile

## Abstract

We analysed whether the hydroethanolic extracts from leaves of *Haplopappus baylahuen* Remy (bailahuen) and *Aloysia citriodora* Palau (cedron) inhibit the growth and ability of *Salmonella* Enteritidis to form biofilms and to adhere to human intestinal epithelial cells. Herein, we first determined the total phenolic content and antioxidant and antibacterial activities of the extracts. Then, *Salmonella* Enteritidis was treated with the extracts to analyse biofilm formation by scanning electronic microscopy and the violet crystal test. We also measured the efflux pump activity of *Salmonella* Enteritidis since biofilm formation is associated with this phenomenon. Furthermore, the human intestinal cell line Caco-2 was infected with *Salmonella* Enteritidis pretreated with the extracts, and 30 min later, the number of bacteria that adhered to the cell surface was quantified. Finally, we determined by qPCR the expression of genes associated with biofilm formation, namely, the diguanilate cyclase AdrA protein gene (*adrA*) and the BapA protein gene (*bapA*), and genes associated with adhesion, namely, the transcriptional regulator HilA (*hilA*). The phenolic content and antioxidant and bactericide activities were higher in bailahuen than in the cedron extract. Biofilm formation was inhibited by the extracts in a dose-dependent manner, while the activity of efflux pumps was decreased only with the cedron extract. Adhesion to Caco-2 cells was also inhibited without differences between doses and extracts. The extracts decreased the expression of *adrA*; with the cedron extract being the most efficient. The expression of *hilA* is affected only with the cedron extract. We concluded that hydroethanolic extracts of bailahuen and cedron differentially inhibit the growth of *Salmonella* Enteritidis and affect its the ability to form biofilms and to adhere to human intestinal epithelial cells. These results highlight the presence of molecules in bailahuen and cedron with a high potential for the control of the *Salmonella* Enteritidis pathogenesis.

## 1. Introduction

*Salmonella enterica* subspecies *enterica* is a worldwide common pathogen that causes foodborne diseases [1]. The *enterica* subspecies is subdivided into two groups: typhoid and nontyphoid strains [2]. Some reports indicate that nontyphoid strains are a growing global problem because of the low health standards in many countries around the world and poor clinical follow-up in underdeveloped countries [1]. From 2013 to 2014, the number of salmonellosis cases in the European Union increased by 19%, being more prevalent in children between 0 and 4 years old than in adults. Likewise, the hospitalization rate of confirmed cases was 77-85% [3, 4]. In Africa, a high prevalence of invasive salmonellosis has been observed in people with HIV and malaria, and also in children under 5 years old with an estimated fatality rate of 20.6% [3]. The costs associated with salmonellosis are estimated in 3,309,000 US dollars, making it the pathogen that generates the most costs [5]. In Chile, the Institute of Public Health (ISPCH) reported that 74% of outbreaks of foodborne diseases in 2015 were caused by *Salmonella* infections [6]. From the two serotypes that cause nontyphoid disease, *Salmonella* Enteritidis is the predominant strain in most countries, and the most common in clinical isolates [1, 7].

The poultry industry represents the most important reservoir for *Salmonella* Enteritidis; this pathogen is able to form biofilms on the production equipment and the poultry's gut. The presence of biofilms of pathogenic microorganisms in the food industry has a serious risk for human health [8–10]. Biofilm formation is an environmental resistance mechanism that allows pathogens to settle on biotic or abiotic surfaces through an exopolysaccharide matrix, which confers resistance and permanence on the surface. The CsgD protein (Curlin subunit gene D) is the most important transcriptional regulator in the formation of biofilms because it regulates the transcription of the Curli complex and activates the production of the protein BapA, which is fundamental in cell aggregation [11]. CsgD also activates AdrA expression (*diguanilato cyclase AdrA*), which is involved in cellulose synthesis necessary for biofilm formation [12, 13]. Recently, interest has been growing on the role of expulsion pumps in the formation of biofilms. These pumps, which play a key role in antibiotic resistance, are removed from the inside of the cell, preventing their therapeutic action [14]. Studies in mutants with deletions of efflux genes decreased biofilm formation in *Salmonella* Typhimurium [15], elucidating the importance of efflux pumps as possible therapeutic targets in the environmental eradication of *Salmonella*. After the ingestion of contaminated food, *Salmonella* adheres into the intestinal epithelium via adhesions of its fimbriae, which apparently bind to membrane proteins, glycosylated residues, or lipid structures [16, 17]. In order to adhere to epithelial cells, *Salmonella* converts to adhesion protein SiiE (giant nonfimbrial adhesion protein), which is essential to establish cell contact that allows the translocation of effector proteins facilitating the entry of *Salmonella* [18]. The SiiE expression is regulated by HilA, and previous studies indicate that *Salmonella* Enteritidis mutants *∆hilA* showed a reduced ability to adhere to and invade the human intestinal epithelial cell line Caco-2 [19].

New compounds with properties that inhibit the formation of biofilms and with the ability to adhere to intestinal epithelial cells could be a good alternative to decrease *Salmonella* infective capacity. In this context, Almeida et al., in 2018, tested in silico 107 common compounds in plants and their effect on biofilms, and it was observed that 83.2% of the compounds were able to inhibit the formation of *Salmonella* Enteritidis biofilms. Most of these compounds correspond to flavonoids, methoxyphenols, and monoterpenes. There is evidence on the inhibitory effects of some secondary plant metabolites in sublethal doses on biofilm formation of *Salmonella*. The berberine alkaloid inhibits biofilm formation at a concentration of 0.625 mg/ml [20]. Some metabolites isolated from grape extract, such as gallic acid (52%), cinnamic acid (51%), and vinyl acid (49%), at concentrations of 100 g/ml also have an inhibitory effect on biofilm formation of *Salmonella* Typhi [21]. *Haplopappus baylahuen* (bailahuen) and *Aloysia citriodora* Palau (cedron) are common plants in South America with medicinal properties including treatment for stomach and liver ills [22]. Extracts and infusions of *Haplopappus baylahuen* have a high antioxidant capacity [23], while cedron essential oils have shown extensive biological activity, such as antioxidant, anticancer, antimicrobial, anesthetic, and sedative activities, in in vitro and in vivo studies [24]. These characteristics encourage us to investigate whether these plants are useful for inhibiting the virulence of enteropathogens. Herein, we analysed the content of total phenols, antioxidant activity, and bactericide effect of the bailahuen and cedron hydroethanolic extracts. Then, *Salmonella* Enteritidis was treated with sublethal doses of the extracts to determine whether they are able to inhibit the ability of the bacteria to form biofilms and its association with the efflux pump activity and expression of *adrA* and *bapA*. Finally, the effects of the extracts on adhesion of *Salmonella* Enteritidis to the human intestinal cell line Caco-2 and its association with *hilA* were determined.

## 2. Materials and Methods

### 2.1. Recollection of the Plants and Preparation of the Hydroethanolic Extracts

First, we proceeded to collect the plant material (Table 1). The plants were identified as *Haplopappus baylahuen* (bailahuen) and *Aloysia citriodora* Palau (cedron) and provisionally deposited in the Department of Biology of Universidad de Santiago de Chile with the registration number of 001/S1 and 002/S1, respectively. The plants were dried at 35°C for 24 hours. Subsequently, the extraction of dry leaves (3 g) was made with 100 ml ethanol (85% *v*/*v*). The samples were sonicated for 60 minutes and macerated for 72 hours, filtered, and dried at 37°C and then stored at 4°C until use. The hydroethanolic extracts were diluted with dimethyl sulfoxide (DMSO) 0.1% as vehicle.

### 2.2. Measurement of Total Phenolic Content and Antioxidant Activity of the Hydroethanolic Extracts of Bailahuen and Cedron

The total phenols were determined using the Folin-Ciocalteu method [25]. One *μ*l (0.5 mg/ml) of the hydroethanolic extracts was added to 99 *μ*l of distilled water and then mixed with 100 *μ*l of the Folin-Ciocalteu reagent. After 2 minutes of incubation under darkness, 800 *μ*l of Na_2_CO_3_ was added and incubated for an additional of 20 minutes at 40°C. Absorbance was measured using a spectrophotometer (SmartSpec™ 3000) at 740 nm. The total phenolic content was calculated as gallic acid equivalent (*μ*g Ac. eq/ml). We used 0.5 mg/ml of extract because the reagent reacts completely at this concentration. This assay was repeated six times.

To determine the antioxidant activity, we used the method based on Brand-Williams et al. in 1995 [26] with some modifications. Briefly, 2 *μ*l of the hydroethanolic extract at 0.5 mg/ml was mixed with 100 *μ*l of 2,2-diphenyl-picrylhydrazyl (DPPH) 150 *μ*M. Then, the samples were incubated for 2 hours at room temperature and darkness, and the absorbance was read using a spectrophotometer at 515 nm. This assay was repeated six times.

### 2.3. Determination of the Bactericide Activity of the Hydroethanolic Extracts of Bailahuen and Cedron

*Salmonella enterica* subs. *enterica* serovar Enteritidis ATCC13076 was used to determine the bactericide activity as well as the Minimal Inhibitory Concentration (MIC) of the hydroethanolic extracts. The MIC was determined in the Mueller-Hinton broth medium with the microdilution method in 96-well plates [27, 28]. In this assay, we used a concentration of extract ranging between 0.25 and 20 mg/ml dissolved in the Mueller-Hinton broth and with a bacterial suspension of 1 × 10^8^ UFC/ml to 200 *μ*l final volume. We considered as MIC the lowest concentration where the inhibition of bacterial growth is total [29, 30]. Streptomycin (Sigma-Aldrich®) was used as a positive control.

### 2.4. Antibiofilm Activity of the Hydroethanolic Extracts of Bailahuen and Cedron

We incubated 10 *μ*l of *Salmonella* Enteritidis ATCC13076 adjusted to 1 × 10^8^ bacteria/ml with sublethal concentrations of the extracts at 1 and 2 mg/ml in LB broth under 24-well plates with cover glasses. The vehicle treatment is LB broth and DMSO. After 24 hours of incubation, the covers were fixed with heat, stained with safranin (0.1%), and dried at 35°C overnight; then, the covers were mounted on brackets, sputtering with a golden bath and observed using an scanning electron microscope (Zeiss EVO MA10). To quantify the inhibition of a biofilm, a violet crystal test in a 96-well plate was performed. The function of this assay is to determine the ability of the extract to inhibit the biofilm formation of *Salmonella* in a polypropylene plate. Briefly, 100 *μ*l of LB broth with 1 and 2 mg/ml of extract and 10 *μ*l of bacteria cultures adjusted at a concentration of 1 × 10^6^ bacteria/ml was added into sterile 96-well polystyrene plates and incubated at 37°C for 24 hours. Then, the biofilm was fixed with methanol 99%, washed with PBS and dyed with crystal violet 0.01% for 15 minutes, washed thrice, and finally 100 *μ*l of acetic acid 30% was added. The plate with the crystal violet suspension was measured using a Tecan Infinite PRO at 550 nm [31, 32].

### 2.5. Effect of the Hydroethanolic Extracts of Bailahuen and Cedron on Efflux Pump Activity of *Salmonella* Enteritidis

A method used primarily to determine the effect of substances on pumps of the RND type (resistance-nodulation-division family transporters) was used. *Salmonella* Enteritidis ATCC13076 were suspended in LB broth overnight and then centrifuged, washed, and resuspended in 0.9% saline solution with a final concentration of 2 mg/ml of extract and incubated in a plate of 96 wells at 30°C for 30 minutes; the inoculum was adjusted to 1 × 10^8^ UFC/ml. Then, 0.4% glucose and 0.5 g/ml ethidium bromide were added. The plate was measured every 5 minutes for 50 minutes at 520 and 590 nm of excitation and emission, respectively. The equipment used was a TECAN Infinite PRO [16, 24]. The fluorescence of the ethidium bromide remains stable when the pumps operate normally because there is an equilibrium between the input and output of ethidium bromide occurring across the pumps. When a substance inhibits efflux pumps, fluorescence increases due to the accumulation of the substrate inside the bacteria. In contrast, a substance stimulates the pumps when the fluorescence values decay overtime.

### 2.6. Effect of the Hydroethanolic Extracts of Bailahuen and Cedron on Adhesion of *Salmonella* Enteritidis into Caco-2 Cells

For this assay, we used 24-well plates with coverslips (12 mm) seeded with 3 × 10^5^ cells/well of the cell line derived from human intestinal carcinoma, Caco-2. *Salmonella* Enteritidis ATCC13076 were cultivated in LB broth for 18 hours at 37°C and treated with 1 and 2 mg/ml of extract. The next day, bacteria were centrifuged, washed, and adjusted to 3 × 10^7^ bacteria/ml in the DMEM medium giving a multiplicity of infection (MOI) of 100 to infect Caco-2 cells for 30 minutes at 37°C and 5% CO_2_. Then, the cells were washed three times with PBS and fixed with formaldehyde 4% for 10 minutes and stained for 5 minutes with safranin 0.05%; the covers were again washed until colour is not detached and mounted on a slide with Canada balsam and observed in a light microscope (Zenith Lab Inc. XSZ-107BN). To perform a quantitative analysis, the same assay was replicated with some variations. The bacteria were pretreated with 1 and 2 mg/ml of extracts and infected the Caco-2 cell line with MOI of 100. After incubation, the covers were rinsed 5 times with PBS and treated with Triton X-100 0.1% to determine the colony-forming unit (CFU). The initial and final CFU numbers were normalized to the adhesion capacity of the bacteria without treatment.

### 2.7. Effect of the Hydroethanolic Extracts of Bailahuen and Cedron on the Expression of *adrA*, *bapA*, and *hilA* Genes of *Salmonella* Enteritidis

*Salmonella* Enteritidis ATCC13076 were cultured in LB medium with 1 mg/ml of the extracts. After 24 hours, the bacteria were centrifuged at 12000 g for 10 minutes, and then the RNA was extracted using 600 *μ*l TRIzol™ Reagent (Invitrogen™) as recommended by the manufacturer. The cDNA was synthesized using the enzyme RevertAid H Minus Reverse Transcriptase (Thermo Scientific™). With the cDNA, rt-qPCR analysis was made on the AriaMx Real-time PCR System (Agilent Technologies) thermocycler with the Takyon™ ROX SYBR® MasterMix dTTP Blue (Eurogentec) kit using the primers described in Table 2. As housekeeping gene, we use 16S rRNA and the relative expression levels were calculated using the 2^−ΔΔCt^ method.

### 2.8. Statistical Analysis

The results are expressed as percentage of the corresponding control (means ± standard deviation). Statistical analyses were performed with the program GraphPad Prism 5.01 (San Diego, CA). The statistical difference of total phenolic content and antioxidant and antibiofilm activities, and the effect on efflux pump activity and on the expression of a*drA*, *bapA*, and *hilA* genes were carried out by employing one-way analysis of variance (ANOVA) followed by Bonferroni's post hoc multiple comparison test at a significance level of *p* < 0.05. The MIC and antiadhesion activity are analysed using the Kruskal Wallis test and the Dunn test.

## 3. Results and Discussion

### 3.1. Content of Total Phenols and Antioxidant Activity in the Hydroethanolic Extracts of Bailahuen and Cedron

The results of the Folin-Ciocalteu assay showed that the extract of bailahuen which had a total phenol content of 3899.4 ± 594.6 *μ*g of gallic acid equivalent/ml was significantly different when compared to that of the cedron extract which had 1898.3 ± 347.0 *μ*g of gallic acid equivalent/ml (*p* < 0.05). On the other hand, the antioxidant activity of the extract expressed as inhibition of DPPH percent showed that the bailahuen extract inhibits by 74.5 ± 4.8% and the cedron extract inhibits by 40 ± 7.8%, indicating that bailahuen is a better antioxidant than cedron (*p* < 0.05). The antioxidant activity of plant extracts is an essential feature to predict their phytomedicinal attributes. Our results corroborate previous reports showing a direct correlation between the phenol content and the antioxidant capacity of fruits (berry) and culinary (oregano) and medicinal herbal extracts (peppermint, valerian) [33–38]. Phenolic compounds are important antioxidant components that are responsible for deactivating free radicals based on their ability to donate hydrogen atoms to free radicals. Therefore, it is probable that both extracts of bailahuen and cedron contain phenolic compounds that may be useful to develop new pharmacological agents against different human pathologies, including *Salmonella* Enteritidis infection.

### 3.2. The Extracts of Bailahuen and Cedron Have Bactericide Activity for *Salmonella* Enteritidis

We found that MIC was from 9 ± 1.5 mg/ml for cedron to 11 ± 1.5 mg/ml for bailahuen (Table 3); this value is statistically different (*p* < 0.05). The MIC of bailahuen and cedron are high compared to other extracts such as those of *Mitracarpus frigidus* [29], which has a 1 mg/ml MIC, or those of red seaweed extracts capable of significantly reducing growth to 2 mg/ml in *Salmonella* Enteritidis [39]. This indicates that the extracts of bailahuen and cedron have components with low bactericide capacity compared with extracts of other medicinal plants.

Establishing the minimum inhibitory concentrations allowed us to determine the sublethal doses (1 and 2 mg/ml) to analyse the effects of the extracts on the formation of biofilms and adhesion of *Salmonella* Enteritidis regardless of growth inhibition.

### 3.3. Bailahuen and Cedron Extracts Inhibit Biofilm Formation in *Salmonella* Enteritidis

**Figure 1:**
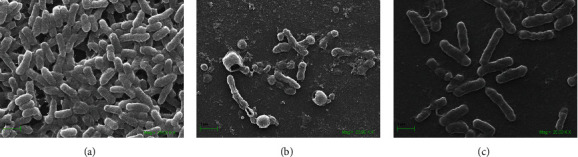
*Salmonella* Enteritidis biofilm scanning electron microscopy. Formation of biofilms of *Salmonella* Enteritidis in plates of 24 wells with coverslips treated with extracts of bailahuen and cedron at 1 mg/ml observed under a scanning electron microscope. (a) Vehicle, (b) bailahuen, and (c) cedron. Note that all extracts show inhibition in the biofilm formation in contrast with the control.

**Figure 2:**
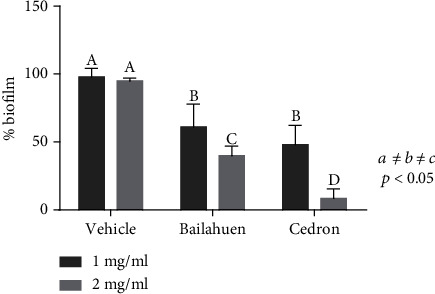
Biofilm quantification. Crystal violet assay in 96-well plates with *Salmonella* Enteritidis incubated with vehicle (control) or 1 or 2 mg/ml of bailahuen or cedron extract. After 24 hours of incubation, the biofilms were fixed with methanol and dyed with violet crystal. The absorbance was then measured at 550 nm. The data are shown as percentage of control (mean ± standard deviation; *n* = 6). The values *a* ≠ *b* ≠ *c* ≠ *d* indicate significant difference, *p* < 0.05. Note that the two extracts decrease biofilms, although cedron was more effective.

In the vehicle group, a dense biofilm formed by multilayers of bacteria tightly linked together was observed (Figure 1(a)), while treatment with bailahuen (Figure 1(b)) induced morphological changes in the bacteria, induced great damage to the cell walls, and induced changes in size, inhibiting biofilm formation. In the cedron extract (Figure 1(c)), the image shows less damage than that shown in Figure 1(b) and also decreased size and inhibition in the biofilm formation. The quantitative analysis indicated that at the concentration of 1 mg/ml, the biofilm formation was 42.8 ± 7.5% and 51.9 ± 6.3% for bailahuen and cedron, respectively, while at the concentration of 2 mg/ml, the biofilm formation was 8 ± 8.6% and 40 ± 3.0% for cedron and bailahuen, respectively (Figure 2). This shows statistical difference (*p* < 0.05). All treatments with the extracts were statistically different with the control condition (*p* < 0.05). The quantification results are directly related to those observed in the scanning electron microscope images. It is interesting to note that the extract with less antioxidant capacity is the most effective extract in inhibiting biofilm formation. This situation could be related to the type of compounds present, rather than the amount. There are multiple reports on extracts with antioxidant activity that demonstrate their antibiofilm effect in different microorganisms [40–45], but these works did not show any correlation between the percentage of antioxidant activity and the inhibition of biofilm formation. To establish a pattern or correlation, it is necessary to perform more analysis with different extracts and with isolated molecules.

### 3.4. The Cedron Extract, but Not Bailahuen, Decreased Efflux Pump Activity of *Salmonella* Enteritidis

**Figure 3:**
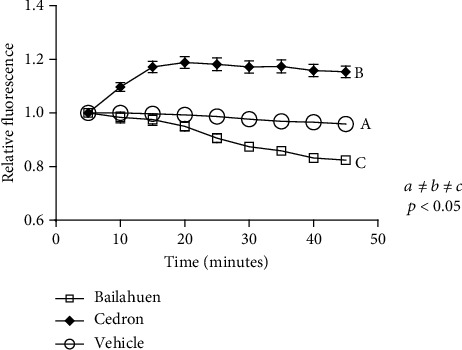
Fluorescence of the ethidium bromide in *Salmonella* Enteritidis. Intracellular accumulation of ethidium bromide in *Salmonella* Enteritidis pretreated with bailahuen and cedron. *Salmonella* Enteritidis were incubated in 96-well plates with vehicle (control) or 1 or 2 mg/ml of bailahuen or cedron extract; then, each was measured every 5 minutes for 50 minutes at 520 and 590 nm of excitation and emission, respectively. The data are shown as percentage of control (mean ± error bars; *n* = 5). The values *a* ≠ *b* ≠ *c* indicate significant differences, *p* < 0.05.

The cedron extract inhibits the functioning of the RND pumps between 5 and 20 minutes, while the bailahuen extract stimulated efflux pump activity at 25 and 50 minutes (Figure 3), with significant differences observed between them and the control condition (*p* < 0.05); these results indicate that only cedron utilizes this mechanism to decrease biofilm formation in *Salmonella* Enteritidis. Paradoxically, the bailahuen extract increased efflux pump activity although it also inhibited biofilm formation of *Salmonella* Enteritidis. This could indicate that the effect of bailahuen on biofilm formation is not mediated by the inhibition of RND efflux pump activity; probably, other efflux pump families or other mechanisms that bind to adhering proteins or cellulose synthesis of the bacteria could be involved in the effect of bailahuen on biofilm formation, but this remains to be proven. The results obtained with cedron could be used to study a possible synergy of this extract with antibiotics in the treatment of multiresistant bacteria. If cedron can inhibit efflux pumps for a certain time, this could mean that antibiotics can be effective before being expelled from the bacteria.

### 3.5. Bailahuen and Cedron Hydroethanolic Extracts Reduce the *adrA* Gene in *Salmonella* Enteritidis

**Figure 4:**
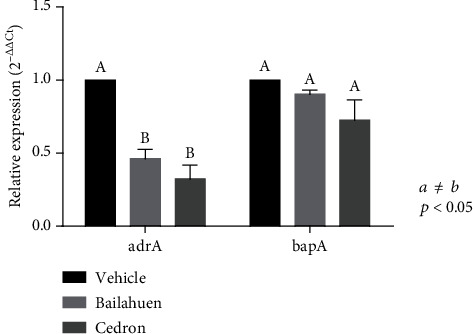
Quantification of the relative expression the *adrA* and *bapA* genes. *Salmonella* Enteritidis were incubated with vehicle (control) or 1 or 2 mg/ml of bailahuen or cedron extract for 24 hours; subsequently, RNA was extracted, and cDNA was formatted to determine gene expression levels using qPCR. The data are shown as percentage of control (mean ± error bars; *n* = 6). The expression of *adrA* decreased by more than 50% in the presence of bailahuen extract. Extracts did not affect the expression of the *bapA* gene. The values *a* ≠ *b* indicate significant difference, *p* < 0.05.

Bailahuen and cedron extracts reduced the expression of the *adrA* gene by 46.4 ± 6.1% and 32.4 ± 9.6%, respectively, being distinctly different from the control condition (*p* < 0.05). Interestingly, the extracts of bailahuen and cedron did not change the expression of the *bapA* gene (Figure 4).

Studies in mutant *Salmonella* with low or no expression of *adrA* showed the formation of thin and fragile biofilms compared to the normal condition [46]. Results were also similar to those obtained in this investigation, observing the images of SEM; thus, we suggest that one of the pathways used by extracts to decrease the formation of biofilms is by *adrA*.

On the other hand, our results show that the extracts of bailahuen and cedron did not change the expression of the *bapA* gene suggesting that this pathway is not relevant in the effects of the extracts on biofilm formation. These results only indicate that there is no effect on gene transcription, but it is necessary to analyse the effect of extracts to the BapA protein at the posttranslational level.

### 3.6. Bailahuen and Cedron Extracts Inhibit Adhesion of *Salmonella* Enteritidis into the Caco-2 Cell Line

**Figure 5:**
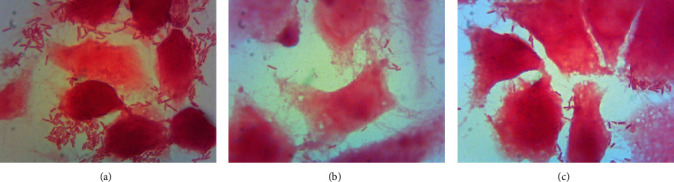
*Salmonella* Enteritidis adhesion to Caco-2 cells. Optical microscope image (100x) showing bacteria that adhered to Caco-2 cells (*n* = 4). The bacteria were treated with vehicle (a), 1 mg/ml of bailahuen (b), and cedron (c) extracts 18 h before infection of the cells.

**Figure 6:**
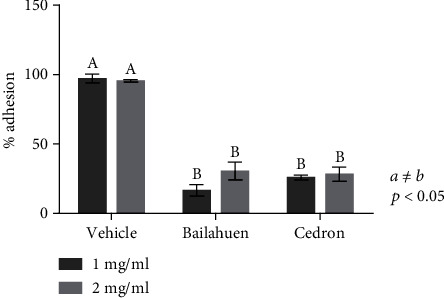
Quantification of adhesion of *Salmonella* Enteritidis to Caco-2 cells. Percentage of adhesion to Caco-2 cells of bacteria pretreated with vehicle (control) or 1 or 2 mg/ml of bailahuen or cedron extract. The data are shown as percentage of control (mean ± error bars; *n* = 5). The values *a* ≠ *b* indicated significant difference, *p* < 0.05. Note that both extracts decrease the ability to adhere to *Salmonella* Enteritidis epithelial cells.

There was a greater number of bacteria that adhered to the cell surface in the vehicle (Figure 5(a)) than in the groups treated with bailahuen or cedron extracts (Figures 5(b) and 5(c)). When we evaluated the quantification of *Salmonella* Enteritidis adhesion to Caco-2 cells (Figure 6), we found that in the bailahuen group, the adhesion to the host cell was reduced by 21.3 ± 4.1% at 1 mg/ml and 30.5 ± 6.3% at 2 mg/ml. On the other hand, in the cedron group, the adhesion to the host cell was decreased by 26.8 ± 1.6% at 1 mg/ml and 28.3 ± 5.0% at 2 mg/ml. We note that there are significant differences in all treatments with respect to the control (*p* < 0.05), but there are no statistical differences between cedron and bailahuen.

Our results are concordant with previous reports showing that phenolic compounds reduce the adhesion and invasion capacity of *Salmonella*. Carvacrol, present in oregano oil, decreased the adhesion and invasion of *Salmonella* Typhimurium in Caco-2 and IPEC-J2 cells [47]. Furthermore, extracts of *Chondrus crispus* and *Sarcodiotheca gaudichaudii* decreased *Salmonella* Enteritidis' ability to colonize various tissues such as the ceca, spleen, liver, and ovary in birds [48]. Thus, the extracts of bailahuen and cedron could have similar chemical compounds as those of the other plant extracts, which efficiently decrease adhesion of *Salmonella* to its host cells. The results obtained in the two assays are associated, and we observed a reduction in the number of bacteria that remain attached to the surface after incubation and subsequent washing to remove the planktonic cells.

### 3.7. Bailahuen and Cedron Extracts Reduce the *hilA* Expression in *Salmonella* Enteritidis

**Figure 7:**
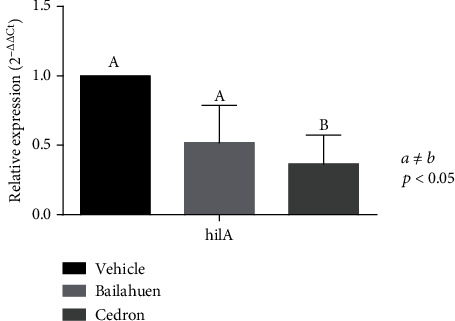
Quantification of the relative expression of the *hilA* gene. *Salmonella* Enteritidis was incubated with vehicle (control) or 1 or 2 mg/ml of bailahuen or cedron extract for 24 hours; subsequently, RNA was extracted, and cDNA was formatted to determine gene expression levels using qPCR. The data are shown as percentage of control (mean ± error bars; *n* = 6). The values *a* ≠ *b* indicate significant difference, *p* < 0.05. Note that the expression of *hilA* decreases by more than 50% in the presence of bailahuen and cedron extracts with respect to the control.

The expression of the *hilA* gene in *Salmonella* Enteritidis treated with the extracts of cedron decreased 63.4 ± 11.9% with respect to vehicle (*p* < 0.05) (Figure 7). On the other hand, the bailahuen extract reduced the expression of *hilA* at 49 ± 15.5%, but it was not statistically different.

There is evidence that berry extracts and red seaweeds of *Sarcodiotheca gaudichaudii* and *Chondrus crispus* decrease bacterial adhesion by repressing genes like *invA* (invasion protein), *invF* (invasion regulatory protein), *sirA* (transcriptional regulator), or *sirB* (transcriptional factor) [33, 39]. In this work, we show that cedron reduces the expression of *hilA* suggesting that this extract utilizes the *hilA* pathway to inhibit adhesion of *Salmonella* Enteritidis to Caco-2 cells. The observed differential effects of the different types of plants on the genes involved in the bacteria adhesion could be explained by differences in the content of metabolites or molecular structures specifically present in each plant. On the other hand, since the bailahuen extract also reduced the adhesion capacity of *Salmonella* Enteritidis but did not affect expression of *hilA*, we can speculate that other mechanisms that control the adhesion of *Salmonella* Enteritidis could be involved with the effect of bailahuen. Further studies are necessary to quantify other genetic markers and thus determine the mechanism by which the bailahuen extract decreases the adhesion of *Salmonella* Enteritidis to Caco-2 cells. Identification of molecular structures present in bailahuen and cedron could provide more details concerning the differential effects on the biofilm formation or adhesion to host cells of *Salmonella* Enteritidis, but this was not done.

## 4. Conclusions

In this work, we showed that the extracts of cedron and bailahuen differ in their content of total phenols and antioxidant activity. Furthermore, both extracts inhibit growth of *Salmonella* Enteritidis at higher concentrations than other plant extracts. However, lower concentrations of bactericide activity differentially affected the ability of *Salmonella* Enteritidis to form a biofilm and adhere to human intestinal epithelial cells. The antioxidant activity is directly proportional to the total phenol content, indicating that the phenolic compounds present in the extracts are effective antioxidants and free radical inhibitors. We show that bactericide activity is not proportional to phenol content or antioxidant activity since cedron is most effective for inhibiting growth although it is the extract with a lower antioxidant activity. This indicates that there are phenolic compounds in the cedron extract with bactericide activity but not with antioxidant activity. The extracts of bailahuen and cedron can inhibit the ability of *Salmonella* Enteritidis to form a biofilm and adhere to human intestinal cells. In the case of the inhibition in biofilm formation, the cedron extract proved to be the most effective. This effect could be explained by the inhibition of efflux pump activity and the *adrA* signalling pathway. The inhibition of the ability of *Salmonella* Enteritidis to adhere to the surface of epithelial cells was efficient with all extracts. However, this effect probably is associated to the decreased *hilA* expression only in the cedron extract. Considering that biofilm formation and adhesion to host cells allow the survival of *Salmonella* in the environment and in the intestine of poultry, we propose that these extracts alone or combined could be used in the poultry industry as part of the feeding of the birds. This also can reduce the intestinal load of the animals and thus decrease the contamination, avoiding the selection of resistant microorganisms and the permanence of these in the human population.

## Figures and Tables

**Table 1:** Sites of collection of the vegetal material.

Plant	Site	Coordinates
Bailahuen	San José de Maipo, Santiago, Chile	-33.5896987(lat), -70.3957625 (long)
Cedron	Recoleta, Santiago, Chile	-33.3912008 (lat), -70.6442468 (long)

**Table 2:** Primer sequences.

	Forward	Reverse	Reference
16S rRNA	AGGCCTTCGGGTTGTAAAGT	GTTAGCCGGTGCTTCTTCTG	[13]
hilA	AATGGTCACAGGCTGAGGTG	ACATCGTCGCGACTTGTGAA	[33]
bapA	TTCGAAATGACTGGCGT	GATCATTTAGCGTGAGCT	[13]
adrA	GAAGCTCGTCGCTGGAAGTC	TTCCGCTTAATTTAATGGCCG	[34]

**Table 3:** Minimum inhibitory concentration of ethanolic extracts (*n* = 5).

Treatment	MIC (mg/ml)
Bailahuen	11 ± 1.0
Cedron	9 ± 1.5
Streptomycin	0.003 ± 0.0

## Data Availability

The raw data used to support the findings of this study are available from the corresponding author upon request.

## References

[1] Besser J. M. (2018). *Salmonella* epidemiology: A whirlwind of change. *Food Microbiology*.

[2] LaRock D. L., Chaudhary A., Miller S. I. (2015). Salmonellae interactions with host processes. *Nature Reviews. Microbiology*.

[3] European Food Safety Authority (2014). The European Union summary report on trends and sources of zoonoses, zoonotic agents and food-borne outbreaks in 2012. *EFSA Journal*.

[4] Threlfall E. J., Wain J., Peters T. (2014). Egg-borne infections of humans with salmonella: not only an *S*. *enteritidis* problem. *World's Poultry Science Journal*.

[5] Batz G., Hoffmann M., Morris S. (2011). Ranking the risks: The 10 pathogen-food combination with the greatest burden on public health. *Report of the Emerging Pathogen Institute, University of Florida*.

[6] de EM D., de Chile D. S. G. (2015). Brotes de enfermedades transmitidas por los alimentos situación epidemiológica, Enero—Diciembre 2015 datos provisorios, semanas epidemiológicas 1 a 52 de 2015 definiciones.

[7] Rabsch W., Tschäpe H., Bäumler A. J. (2001). Non-typhoidal salmonellosis: emerging problems. *Microbes and Infection*.

[8] Bohez L., Ducatelle R., Pasmans F., Botteldoorn N., Haesebrouck F., Van Immerseel F. (2006). *Salmonella enterica* serovar Enteritidis colonization of the chicken caecum requires the HilA regulatory protein. *Veterinary Microbiology*.

[9] Hur J., Kim J. H., Park J. H., Lee Y. J., Lee J. H. (2011). Molecular and virulence characteristics of multi-drug resistant *Salmonella* Enteritidis strains isolated from poultry. *Veterinary Journal*.

[10] USDA F (2017). *Livestock and Poultry: World Markets and Trade*.

[11] Steenackers H., Hermans K., Vanderleyden J., De Keersmaecker S. C. J. (2012). *Salmonella* biofilms: an overview on occurrence, structure, regulation and eradication. *Food Research International*.

[12] Jonas K., Tomenius H., Kader A. (2007). Roles of curli, cellulose and BapA in *Salmonella* biofilm morphology studied by atomic force microscopy. *BMC Microbiology*.

[13] Latasa C., Roux A., Toledo-Arana A. (2005). BapA, a large secreted protein required for biofilm formation and host colonization of *Salmonella enterica* serovar Enteritidis. *Molecular Microbiology*.

[14] Martins M., McCusker M., Amaral L., Fanning S. (2011). Mechanisms of antibiotic resistance in *Salmonella*: efflux pumps, genetics, quorum sensing and biofilm formation. *Letters in Drug Design & Discovery*.

[15] Baugh S., Ekanayaka A. S., Piddock L. J. V., Webber M. A. (2012). Loss of or inhibition of all multidrug resistance efflux pumps of *Salmonella enterica* serovar Typhimurium results in impaired ability to form a biofilm. *The Journal of Antimicrobial Chemotherapy*.

[16] de Jong H. K., Parry C. M., van der Poll T., Wiersinga W. J. (2012). Host-pathogen interaction in invasive salmonellosis. *PLoS Pathogens*.

[17] Velge P., Wiedemann A., Rosselin M. (2012). Multiplicity of *Salmonella* entry mechanisms, a new paradigm for *Salmonella* pathogenesis. *Microbiology*.

[18] Barlag B., Hensel M. (2015). The giant adhesin SiiE of *Salmonella enterica*. *Molecules*.

[19] Lv S., Si W., Yu S. (2015). Characteristics of invasion-reduced hilA gene mutant of *Salmonella* Enteritidis in vitro and in vivo. *Research in Veterinary Science*.

[20] Stermitz F. R., Lorenz P., Tawara J. N., Zenewicz L. A., Lewis K. (2000). Synergy in a medicinal plant: antimicrobial action of berberine potentiated by 5'-methoxyhydnocarpin, a multidrug pump inhibitor. *Proceedings of the National Academy of Sciences of the United States of America*.

[21] Ramadan E. M., Abou-Taleb K. A., Galal G. F., Abdel-Hamid N. S. (2017). Antibacterial, antibiofilm and antitumor activities of grape and mulberry leaves ethanolic extracts towards bacterial clinical strains. *Annals of Agricultural Science*.

[22] Hoffmann A. (1998). *Flora silvestre de Chile zona central*.

[23] Schmeda-Hirschmann G., Quispe C., González B. (2015). Phenolic profiling of the South American “baylahuen” tea (Haplopappus spp., Asteraceae) by HPLC-DAD-ESI-MS. *Molecules*.

[24] Bahramsoltani R., Rostamiasrabadi P., Shahpiri Z., Marques A. M., Rahimi R., Farzaei M. H. (2018). *Aloysia citrodora* Pal au (Lemon verbena): A review of phytochemistry and pharmacology. *Journal of Ethnopharmacology*.

[25] Singleton V. L., Rossi J. A. (1965). Colorimetry of total phenolics with phosphomolybdic-phosphotungstic acid reagents. *American journal of Enology and Viticulture*.

[26] Brand-Williams W., Cuvelier M. E., Berset C. (1995). Use of a free radical method to evaluate antioxidant activity. *Lebensmittel-Wissenschaft & Technologie*.

[27] Cockerill F. R., Wikler M. A., Bush K. (2011). Performance standards for antimicrobial susceptibility testing: twenty-first informational supplement. *Report of the Clinical and Laboratory Standards Institute*.

[28] Wafa B. A., Makni M., Ammar S. (2017). Antimicrobial effect of the Tunisian Nana variety *Punica granatum* L. extracts against *Salmonella enterica* (serovars Kentucky and Enteritidis) isolated from chicken meat and phenolic composition of its peel extract. *International Journal of Food Microbiology*.

[29] Campos L. M., Lemos A. S. O., Silva T. P. (2019). *Mitracarpus frigidus* is active against *Salmonella enterica* species including the biofilm form. *Industrial Crops and Products*.

[30] Gehrke I. T. S., Neto A. T., Pedroso M. (2013). Antimicrobial activity of *Schinus lentiscifolius* (Anacardiaceae). *Journal of Ethnopharmacology*.

[31] Jagani S., Chelikani R., Kim D.-S. (2010). Effects of phenol and natural phenolic compounds on biofilm formation by *Pseudomonas aeruginosa*. *Biofouling*.

[32] Hiller C. C., Lucca V., Carvalho D. (2019). Influence of catecholamines on biofilm formation by *Salmonella* Enteritidis. *Microbial Pathogenesis*.

[33] Salaheen S., Jaiswal E., Joo J. (2016). Bioactive extracts from berry byproducts on the pathogenicity of *Salmonella* Typhimurium. *International Journal of Food Microbiology*.

[34] Lamas A., Paz-Mendez A. M., Regal P. (2018). Food preservatives influence biofilm formation, gene expression and small RNAs in *Salmonella enterica*. *LWT*.

[35] Zheng W., Wang S. Y. (2001). Antioxidant activity and phenolic compounds in selected herbs. *Food Chemistry*.

[36] Speisky H., Rocco C., Carrasco C., Lissi E. A., López-Alarcón C. (2006). Antioxidant screening of medicinal herbal teas. *Phytotherapy Research*.

[37] Simirgiotis M. J., Silva M., Becerra J., Schmeda-Hirschmann G. (2012). Direct characterisation of phenolic antioxidants in infusions from four Mapuche medicinal plants by liquid chromatography with diode array detection (HPLC-DAD) and electrospray ionisation tandem mass spectrometry (HPLC-ESI-MS). *Food Chemistry*.

[38] López-Cobo A., Gómez-Caravaca A. M., Švarc-Gajić J., Segura-Carretero A., Fernández-Gutiérrez A. (2015). Determination of phenolic compounds and antioxidant activity of a Mediterranean plant: The case of *Satureja montana* subsp. *kitaibelii*. *Journal of Functional Foods*.

[39] Kulshreshtha G., Borza T., Rathgeber B. (2016). Red seaweeds *Sarcodiotheca gaudichaudii* and *Chondrus crispus* down regulate virulence factors of *Salmonella enteritidis* and induce immune responses in *Caenorhabditis elegans*. *Frontiers in Microbiology*.

[40] Ong K. S., Mawang C. I., Daniel-Jambun D., Lim Y. Y., Lee S. M. (2018). Current anti-biofilm strategies and potential of antioxidants in biofilm control. *Expert Review of Anti-Infective Therapy*.

[41] Papuc C., Goran G. V., Predescu C. N., Nicorescu V., Stefan G. (2017). Plant polyphenols as antioxidant and antibacterial agents for shelf-life extension of meat and meat products: classification, structures, sources, and action mechanisms. *Comprehensive Reviews in Food Science and Food Safety*.

[42] D’Ávila Oliveira B., Rodrigues A. C., Cardoso B. M. I. (2016). Antioxidant, antimicrobial and anti-quorum sensing activities of *Rubus rosaefolius* phenolic extract. *Industrial Crops and Products*.

[43] de Queiroz Aquino-Martins V. G., de Melo L. F. M., Silva L. M. P. (2019). *In vitro* antioxidant, anti-biofilm, and solar protection activities of *Melocactus zehntneri* (Britton & Rose) pulp extract. *Antioxidants*.

[44] Nadaf N. H., Parulekar R. S., Patil R. S. (2018). Biofilm inhibition mechanism from extract of *Hymenocallis littoralis* leaves. *Journal of Ethnopharmacology*.

[45] Efenberger-Szmechtyk M., Nowak A., Czyzowska A. (2020). Plant extracts rich in polyphenols: antibacterial agents and natural preservatives for meat and meat products. *Critical Reviews in Food Science and Nutrition*.

[46] Solano C., García B., Valle J. (2002). Genetic analysis of *Salmonella enteritidis* biofilm formation: critical role of cellulose. *Molecular Microbiology*.

[47] Inamuco J., Veenendaal A. K. J., Burt S. A. (2012). Sublethal levels of carvacrol reduce *Salmonella* Typhimurium motility and invasion of porcine epithelial cells. *Veterinary Microbiology*.

[48] Kulshreshtha G., Rathgeber B., MacIsaac J. (2017). Feed supplementation with red seaweeds, *Chondrus crispus* and *Sarcodiotheca gaudichaudii*, reduce *Salmonella* Enteritidis in laying hens. *Frontiers in Microbiology*.

